# Genomics and Multi-Omics to Guide Clinical Management in Thyroid Cancer

**DOI:** 10.3390/ijms27167464

**Published:** 2026-08-20

**Authors:** Dhoha Dhieb, Kholoud Bastaki

**Affiliations:** Pharmaceutical Sciences Department, College of Pharmacy, QU Health, Qatar University, Doha P.O. Box 2713, Qatar; dhoha.dhieb@qu.edu.qa

**Keywords:** thyroid cancer, multi-omics, precision oncology, targeted therapy

## Abstract

Thyroid cancer comprises a biologically diverse group of tumors initiated by a limited number of recurrent driver alterations, with additional molecular events promoting dedifferentiation, therapeutic resistance, and aggressive clinical behavior. Advances in tumor sequencing have clarified the molecular architecture of papillary, follicular, oncocytic, poorly differentiated, anaplastic, and medullary thyroid carcinomas, and have already changed management in selected settings. Molecular testing improves diagnostic refinement and risk assessment in cytologically indeterminate thyroid nodules, while alterations involving *BRAF*, *RET*, and *NTRK* can guide targeted therapy in advanced disease. Beyond DNA, transcriptomic, proteomic, epigenetic, metabolomic, immune, spatial, and liquid-biopsy approaches offer functional insight into differentiation state, treatment sensitivity, and resistance, although most remain investigational. Their clinical value depends not only on biological plausibility, but on reproducibility, incremental value beyond established clinicopathological variables, and the ability to alter patient management. Computational tools may further support integration of molecular and clinical data, but their usefulness likewise depends on calibration, external validation, interpretability, and demonstration of decision impact. This review synthesizes the genomic and multi-omics determinants of thyroid cancer management across diagnosis, risk stratification, treatment selection, resistance monitoring, and follow-up, and discusses the practical barriers that continue to limit routine implementation, including assay standardization, cost, access, and real-world feasibility. Progress in precision thyroid oncology will depend on robust validation of emerging biomarkers and clear evidence that they improve patient outcomes.

## 1. Introduction

Thyroid cancer is the most common malignancy of the endocrine system worldwide, with an estimated global incidence approaching 586,000 new cases annually and ranking among the ten most common cancers overall [1,2]. Much of the increase in incidence over recent decades reflects greater detection of small, often indolent tumors through high-resolution imaging and fine-needle aspiration (FNA) cytology, although a true increase in advanced and aggressive disease has also been reported in some populations [2,3,4,5]. For most patients, prognosis remains excellent: well-differentiated, localized tumors are highly curable, with disease-specific survival remaining high at five years [6,7]. This favorable overall outcome, however, conceals marked biological and clinical heterogeneity. Poorly differentiated thyroid carcinoma (PDTC), anaplastic thyroid carcinoma (ATC), and advanced medullary thyroid carcinoma (MTC) account for a disproportionate share of morbidity and mortality and continue to expose major limitations in current management [5,6,8,9].

Contemporary thyroid cancer care is therefore defined by a clinical paradox. At one end of the spectrum, many thyroid nodules and low-risk cancers are investigated or treated more aggressively than their biological behavior warrants. At the other, aggressive, dedifferentiated, or metastatic tumors may progress rapidly and require therapeutic decisions within a narrow clinical window. Standard treatment for differentiated thyroid cancer (DTC) remains highly effective in most cases and is based on surgical resection, risk-adapted radioiodine therapy, and thyroid-stimulating hormone (TSH) suppression, with systemic therapy reserved for structurally progressive, symptomatic, or radioiodine-refractory disease [6,7,10]. In practice, this creates two broad management trajectories: radioiodine-sensitive disease, in which thyroid-lineage function is sufficiently preserved for conventional therapy to remain effective, and radioiodine-refractory disease, in which loss of iodine-handling capacity shifts treatment toward systemic and increasingly genotype-directed approaches. This distinction has become central to precision thyroid oncology because molecular testing is most clinically useful when it helps define where a patient lies along this transition.

The modern classification of thyroid neoplasms reflects this biological diversity. The 2022 World Health Organization (WHO) classification integrates histopathology with cellular lineage, proliferative activity, and increasingly relevant molecular features [11,12,13]. Follicular-cell-derived tumors include papillary thyroid carcinoma (PTC), follicular thyroid carcinoma (FTC), and oncocytic (Hürthle cell) carcinoma, as well as progressively dedifferentiated entities such as differentiated high-grade thyroid carcinoma (DHGTC), PDTC, and ATC [11,12,13]. Medullary thyroid carcinoma is biologically distinct, arising from parafollicular C cells and frequently associated with activating germline or somatic alterations in *RET* [11,12,13]. These distinctions are clinically important because identical molecular events may have different implications depending on histology, disease stage, differentiation state, and therapeutic context.

Several of the most important clinical decisions in thyroid cancer now sit at the interface between conventional pathology and molecular characterization. One such setting is the evaluation of cytologically indeterminate thyroid nodules, particularly Bethesda III and IV categories, which remain a major source of diagnostic uncertainty [10,14,15]. These nodules are not synonymous with malignancy; their cancer risk varies substantially by local prevalence, cytological interpretation, and patient population, and diagnostic surgery may ultimately reveal benign or indolent disease [10,14,15]. Molecular classifiers can refine this uncertainty, but their value depends on whether they change management in a clinically meaningful way, such as avoiding surgery altogether, altering the extent of initial surgery, or guiding surveillance after a negative result. Another major decision point arises in advanced disease, where delayed recognition of an actionable alteration may result in loss of a therapeutic opportunity, especially in ATC and progressive radioiodine-refractory DTC.

Genomic studies, most notably The Cancer Genome Atlas, have established an unusually clear genotype-phenotype relationship in thyroid cancer compared with many other solid tumors [16]. Approximately 90% of PTCs are initiated by a single, mutually exclusive event activating the mitogen-activated protein kinase (MAPK) pathway, most commonly through *BRAF* V600E, RAS isoforms, or gene fusions involving *RET* and *NTRK* [16]. These drivers are not biologically equivalent. *BRAF* V600E-driven tumors are typically associated with lower thyroid differentiation, reduced expression of iodine-handling genes, lower radioiodine avidity, and higher recurrence risk, whereas RAS-driven tumors more often retain follicular-patterned morphology and differentiation-associated programs [7,16,17]. With progression, additional events accumulate, including *TERT* promoter mutations, *TP53* alterations, phosphatidylinositol 3-kinase-AKT (PI3K-AKT) pathway activation, and disruption of chromatin-remodeling complexes such as switch/sucrose non-fermentable (SWI/SNF), all of which are linked to dedifferentiation, treatment resistance, and adverse outcome [9,18,19,20,21].

These genomic insights have already altered care in selected settings. Molecular testing is now routinely used in many centers for indeterminate thyroid nodules, and advanced thyroid cancers carrying *BRAF* V600E, *RET* alterations, or *NTRK* fusions may benefit from matched targeted therapies [14,15,17,22,23,24]. In ATC, rapid molecular profiling has become especially important because *BRAF* V600E identifies a subgroup that may respond to combined *BRAF* and MEK inhibition, potentially altering both systemic treatment and short-term prognosis [8,22]. In MTC, *RET* testing has dual relevance because it can guide selective RET inhibition in advanced disease while also informing hereditary risk assessment and cascade testing in families [8,25]. At the same time, not all recurrent alterations have equivalent clinical maturity. Some, such as *TERT* promoter mutations, mainly refine prognosis; others remain trial-enabling or translational and do not yet support a direct treatment choice [18,20].

The limits of DNA-based profiling are equally important. In radioiodine-refractory DTC, for example, loss of sodium/iodide symporter (NIS) expression or localization is a key functional determinant of treatment failure, but this phenotype cannot be explained by driver mutation status alone [7,17]. Redifferentiation strategies using MAPK-pathway inhibition have shown that radioiodine uptake can be restored in selected tumors, yet this response is incomplete, variable, and constrained by additional molecular events that stabilize lineage loss [21,26,27,28]. Likewise, targeted therapies can select for secondary resistance mutations, bypass signaling, or broader changes in lineage state and tumor microenvironment. These dynamics increasingly justify serial molecular reassessment, including repeat tissue biopsy or circulating tumor DNA (ctDNA) analysis at progression, particularly in advanced disease where resistance mechanisms may alter subsequent treatment options [29,30,31].

For these reasons, interest has expanded beyond genomic drivers alone to multi-omics approaches that capture tumor function more directly. Transcriptomic, proteomic, epigenetic, metabolomic, immune, and spatial profiling may help explain why tumors with similar initiating mutations differ in differentiation state, treatment sensitivity, and resistance patterns. Some of these layers already provide clinically relevant mechanistic insight, whereas others remain exploratory and require substantial validation before routine adoption. Their value should not be assumed on the basis of biological plausibility alone. A molecular readout becomes clinically meaningful only if it improves a defined decision beyond established clinical, pathological, and genomic variables.

The same principle applies to computational integration. Thyroid cancer management increasingly generates information across multiple domains, including ultrasound, cytology, histopathology, molecular testing, treatment history, and longitudinal monitoring. Computational models may help synthesize these data, but statistical performance alone is insufficient to establish clinical usefulness. Models must be calibrated, externally validated, interpretable, and tested against decision-relevant endpoints, such as reduction in unnecessary FNA or surgery, improved identification of aggressive disease, more accurate selection of targeted therapy, or earlier recognition of resistance.

Against this background, the central challenge in precision thyroid oncology is not simply the expansion of molecular knowledge, but the disciplined determination of which molecular information changes management. This review examines genomic and multi-omics determinants of thyroid cancer across diagnosis, risk stratification, treatment selection, resistance monitoring, and follow-up. Particular attention is given to the distinction between biomarkers that are already embedded in clinical care and those that remain investigational, trial-enabling, or mechanistically informative but not yet decision-changing. It also considers the practical barriers that continue to limit implementation, including assay standardization, turnaround time, cost-effectiveness, reimbursement, equity, and real-world feasibility. The goal is to provide a clinically grounded synthesis of how genomics and multi-omics can guide thyroid cancer management across its full biological spectrum.

## 2. Genomic Determinants of Clinical Management Across Thyroid Cancer Types

Genomic actionability in thyroid cancer is best understood in relation to tumor lineage, differentiation state, and the clinical decision being made. In follicular-cell-derived thyroid cancers, the most important practical distinction is between disease that remains sufficiently differentiated for conventional thyroid-directed therapy and disease that has progressed toward radioiodine refractoriness, dedifferentiation, or aggressive clinical behavior [8,11,16]. In medullary thyroid carcinoma, by contrast, molecular testing primarily defines oncogenic dependency and hereditary risk [32,33,34]. Across these settings, genomic alterations differ markedly in evidentiary maturity: some refine diagnosis or postoperative risk stratification, some identify candidates for approved targeted therapies, and others remain translational or trial-enabling rather than directly treatment-selective. The following sections summarize these determinants by major thyroid cancer type.

### 2.1. Papillary Thyroid Carcinoma

Papillary thyroid carcinoma (PTC), which accounts for approximately 80% of thyroid cancers, remains the best-characterized thyroid cancer subtype at the molecular level. Most cases are initiated by a single, mutually exclusive driver event that activates MAPK signaling. The most common alteration is *BRAF* V600E, which is detected in roughly half of classic and tall-cell PTCs. *RAS* mutations, particularly *NRAS* codon 61 with less frequent *HRAS* and *KRAS* alterations, are more strongly associated with follicular-patterned tumors [8,16]. Rearrangements involving *RET*, *NTRK1*, *NTRK3*, and, more rarely, *anaplastic lymphoma kinase (ALK)* account for a smaller but clinically important subgroup and are particularly enriched in pediatric and radiation-associated tumors [35,36].

These drivers are not biologically equivalent. *BRAF* V600E-mutant tumors usually exhibit lower thyroid differentiation, reduced expression of thyroid-lineage genes, decreased radioiodine avidity, and a higher likelihood of recurrence or radioiodine-refractory behavior [8,16]. By contrast, *RAS*-driven tumors more often retain follicular differentiation and are more likely to remain radioiodine-avid [7,8,17]. The clinical relevance of this distinction extends beyond histological classification. Expression of *SLC5A5*, *TG*, *TPO*, and *TSHR* reflects how intact the thyroid-lineage program remains, and therefore how biologically plausible it is that iodine-handling function can be restored or exploited therapeutically [17]. These markers should not be interpreted as direct treatment-selection assays, but rather as functional readouts of retained differentiation.

Progression beyond conventional PTC biology is usually driven by additional molecular events rather than by the initiating driver alone. Among these, *TERT* promoter mutations are the most clinically mature progression-associated biomarkers. Their coexistence with *BRAF* V600E or *RAS* is associated with higher risk of recurrence, distant metastasis, treatment failure, and disease-specific mortality, and this combined status provides stronger postoperative risk stratification than driver mutation status alone [18,20,37]. Other late events, including alterations in *TP53*, *PIK3CA*, and chromatin-remodeling genes such as *ARID1A*, mainly indicate dedifferentiation and resistance rather than a currently approved treatment choice [9,19,21,38].

Genomic testing has two major clinically validated roles in PTC management. The first is in cytologically indeterminate thyroid nodules, especially Bethesda III and IV categories. Molecular classifiers such as Afirma GSC and ThyroSeq v3 can reduce diagnostic uncertainty, but their clinical utility is highly context dependent [10,14,15]. Negative and positive predictive values vary with local malignancy prevalence, assay failure rates, case mix, and whether noninvasive follicular thyroid neoplasm with papillary-like nuclear features is counted as a malignant endpoint. In practice, a benign or low-risk molecular result may support active surveillance or avoidance of diagnostic surgery, whereas a high-risk result may influence either the decision to operate or the extent of initial surgery. Molecular testing is therefore most useful when interpreted within a local clinical algorithm rather than as a universal binary test. Isolated *RAS* positivity in an indeterminate nodule should also be interpreted cautiously: it is lineage-informative and compatible with follicular-patterned neoplasia, but it does not by itself distinguish benign adenoma, borderline lesions, and invasive carcinoma [14,15].

The second major role of genomic testing in PTC concerns advanced disease. Identification of *BRAF* V600E, *RET* fusions, or *NTRK* fusions can directly affect systemic therapy selection in progressive or refractory tumors [22,23,25]. However, genotype alone does not fully define therapeutic vulnerability. This is particularly evident in redifferentiation strategies aimed at restoring radioiodine uptake. MEK inhibition and combined BRAF/MEK inhibition have shown that radioiodine uptake can be restored in a subset of patients, but the evidence must be interpreted carefully [26,27]. Restoration of tracer uptake, eligibility for therapeutic radioiodine, radiographic response, and durable clinical benefit are distinct endpoints and should not be treated as interchangeable. The selumetinib proof-of-principle study established that redifferentiation is biologically possible, especially in RAS-mutant tumors, but it did not establish routine genotype-guided redifferentiation as standard care [26]. Similarly, BRAF-directed redifferentiation in *BRAF* V600E-mutant disease remains promising but not yet universally applicable, particularly when tumors harbor additional alterations that stabilize lineage loss [27,39].

As targeted therapies enter routine use, longitudinal molecular monitoring becomes increasingly relevant. Acquired resistance in advanced PTC may occur through MAPK pathway reactivation, secondary *RAS* pathway events, loss of *NF1* or *NF2*, or broader chromatin-mediated dedifferentiation [28,29,30,31]. In selected cases, repeat tissue biopsy or circulating tumor DNA analysis at progression may help identify resistance mechanisms and guide trial selection or subsequent targeted approaches [29,30]. At present, this remains a trial-enabling or adjunctive strategy rather than a universal standard.

### 2.2. Follicular and Oncocytic (Hürthle Cell) Carcinomas

Follicular thyroid carcinoma (FTC) accounts for approximately 10% of differentiated thyroid cancers and is molecularly characterized by a largely RAS-like profile. Recurrent alterations include *NRAS*, *HRAS*, and *KRAS* mutations, together with *PAX8::PPARG* fusions in a substantial subset of tumors [8,40,41]. Less frequent events such as *PTEN* loss-of-function, activating *TSHR* mutations, and *EIF1AX* alterations are biologically relevant but are not currently used as independent decision-changing markers [41,42]. As in follicular-patterned PTC, molecular findings in FTC often refine lineage classification more reliably than they define an approved therapy.

Clinically, FTC is notable for its hematogenous metastatic pattern, particularly to bone and lung, and for the prognostic effect of late progression-associated events. Among these, *TERT* promoter mutations are again the most clinically mature biomarkers. Coexisting *RAS* and *TERT* alterations identify a subgroup at particularly high risk of recurrence, distant spread, and short-term disease-specific mortality [18,20,41,43]. Additional combinations, such as *EIF1AX* with *RAS*, may also mark progression-prone disease, but these remain less clinically mature than *TERT*-based stratification [42].

Oncocytic (Hürthle cell) carcinoma is genomically distinct from conventional BRAF- or RAS-driven follicular-cell-derived thyroid cancers. Its defining features include recurrent mitochondrial DNA mutations, especially in complex I subunits, and a characteristic pattern of widespread chromosomal loss with near-haploidization of the nuclear genome [44,45]. These tumors are not primarily driven by the canonical kinase-fusion or MAPK architecture seen in PTC. At present, no standard-of-care treatment-selection biomarker exists for oncocytic carcinoma. Integrative studies have identified candidate metabolic dependencies, including reliance on compensatory aerobic fermentation and redox adaptation, but these remain preclinical or early investigational findings rather than clinically actionable targets [44,45]. Taken together, FTC and oncocytic carcinoma illustrate an important principle in thyroid oncology: not all recurrent genomic findings have equivalent therapeutic implications. Some alterations help define biological subtype, some refine prognosis, and some suggest potential metabolic or lineage-based vulnerabilities, but few currently direct approved targeted therapies in the way seen for *BRAF*, *RET*, or *NTRK* in advanced disease.

### 2.3. Poorly Differentiated, High-Grade, and Anaplastic Thyroid Carcinoma

Poorly differentiated thyroid carcinoma, differentiated high-grade thyroid carcinoma, and anaplastic thyroid carcinoma represent progressive states of follicular-cell-derived tumor evolution rather than wholly separate molecular entities. These tumors usually retain an early MAPK driver, most commonly *BRAF* V600E or *RAS*, but acquire additional late events associated with loss of differentiation, genomic instability, and resistance. Recurrent alterations include *TP53* mutations, *TERT* promoter mutations, *CDKN2A* loss, PI3K-AKT-mTOR pathway activation, and defects in chromatin-remodeling and RNA-splicing genes such as *ARID1A*, *ARID1B*, *SMARCB1*, and *RBM10* [9,19,21,38]. This pattern reflects transition from lineage-constrained disease to aggressive, therapeutically complex tumors with multiple mechanisms of escape.

Among these entities, ATC presents the clearest case for urgent and comprehensive molecular testing. Broad genomic profiling at diagnosis is clinically justified because identification of *BRAF* V600E can immediately alter first-line therapy through combined BRAF and MEK inhibition [8,22]. In selected cases, *RET* fusions, *NTRK* fusions, and rare *ALK* rearrangements may also identify matched therapeutic opportunities [33,46]. In this setting, turnaround time is itself clinically meaningful. Delays in testing can result in loss of a therapeutic window in a disease characterized by rapid local progression, airway compromise, and short survival [8,47].

Even when an actionable alteration is identified, response may be constrained by additional progression-associated biology. Primary or secondary resistance can arise through acquired RAS pathway events, further SWI/SNF loss, activation of PI3K-AKT or YAP/TEAD signaling, or broader lineage plasticity [9,19,21,31]. The tumor microenvironment also becomes increasingly relevant, particularly in ATC, where immune exclusion, macrophage-rich infiltrates, and epigenetically shaped immune escape may limit treatment response. These tumors therefore exemplify why genomic profiling must be interpreted alongside tumor state rather than as a simple mutation-to-drug mapping exercise.

Dynamic monitoring at progression may be especially useful in advanced PDTC and ATC, where repeat tissue biopsy is often difficult because of anatomical constraints or rapid deterioration. In high-burden disease, circulating tumor DNA can detect driver and resistance alterations with sensitivity approaching that of tissue sequencing and may reveal evolving resistance before radiographic progression becomes obvious [29,30,31]. However, its routine adoption remains limited by assay standardization, cost, and reduced sensitivity in low-volume disease.

### 2.4. Medullary Thyroid Carcinoma

Medullary thyroid carcinoma (MTC) is biologically distinct from follicular-cell-derived thyroid cancers because it arises from parafollicular C cells and is driven primarily by aberrant RET signaling [32,33,34]. Approximately one quarter of cases are hereditary and occur in the context of multiple endocrine neoplasia type 2, caused by germline activating *RET* mutations. Most sporadic tumors also harbor *RET* alterations, most commonly involving codon M918, whereas a smaller subset carries *RAS* mutations [32,34]. Unlike PTC and FTC, MTC is intrinsically not a radioiodine-responsive disease, and molecular testing is therefore focused on hereditary risk and systemic therapy selection rather than on thyroid-lineage function.

*RET* testing is a standard-of-care requirement at diagnosis for two distinct reasons. First, it identifies candidates for selective RET inhibition in advanced disease, where agents such as selpercatinib and pralsetinib have produced substantial and durable responses in molecularly defined patients [48,49]. Second, germline *RET* testing guides hereditary risk assessment, family counseling, and cascade screening [32,33,34]. These two functions should be conceptually separated: somatic testing informs treatment selection, whereas germline testing informs inherited cancer predisposition and preventive management.

**Figure 1:**
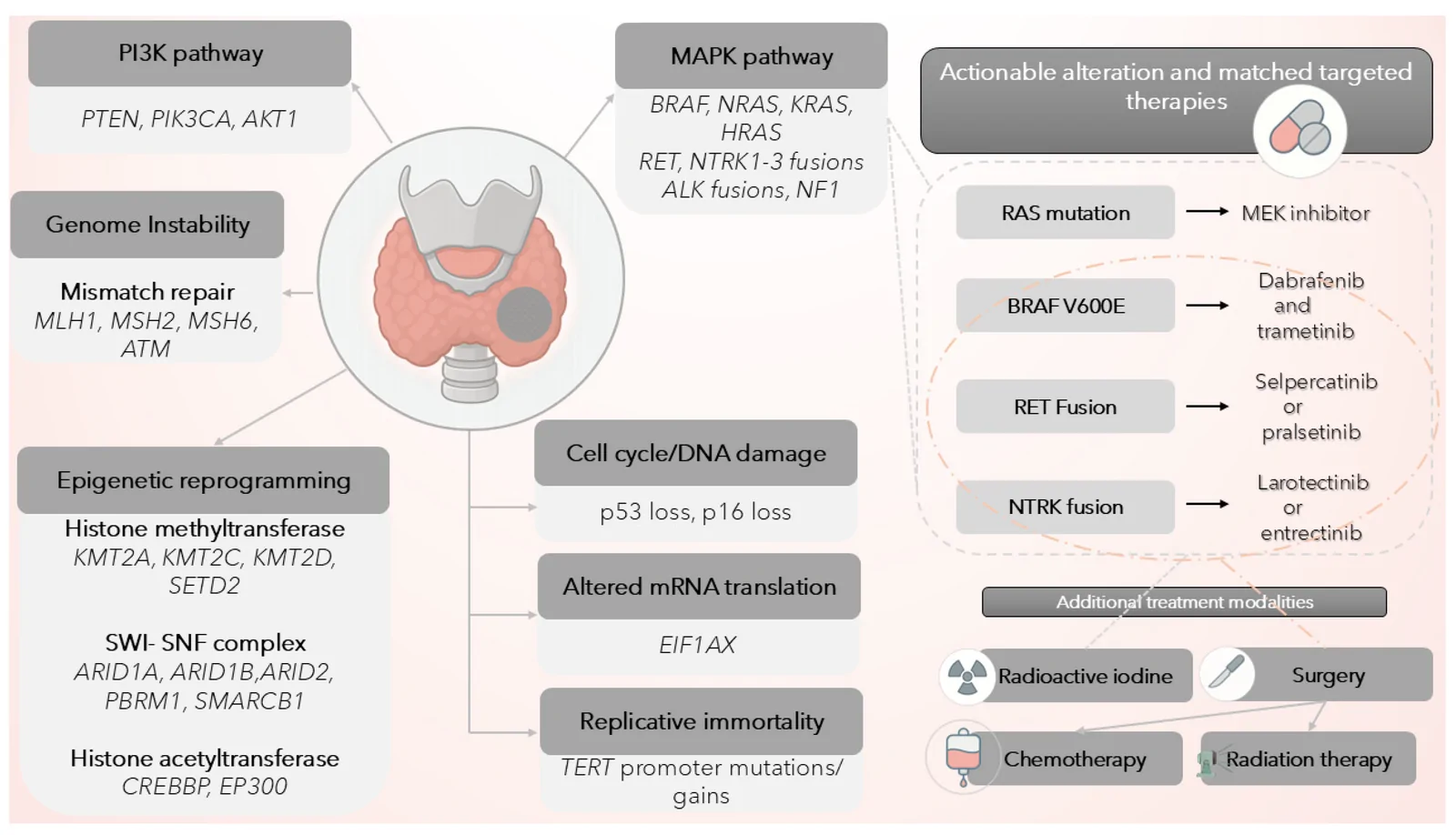
Major genomic drivers, progression-associated alterations, and matched targeted therapies across thyroid cancer subtypes. Follicular-cell-derived thyroid cancers are mainly initiated by MAPK-pathway alterations, including *BRAF*, *RAS*, and kinase fusions involving *RET* and *NTRK*. Progression toward poorly differentiated or anaplastic disease is associated with additional alterations affecting *TERT*, *TP53*, *PI3K-AKT* signaling, chromatin remodeling, genome stability, and RNA translation. The most established matched therapies include BRAF/MEK inhibition for *BRAF* V600E-mutant ATC, selective RET inhibition for RET-altered disease, and TRK inhibition for *NTRK* fusion-positive thyroid cancers.

Resistance to selective RET inhibition occurs in a clinically relevant minority of patients and may involve on-target non-gatekeeper alterations, including solvent-front or hinge-region mutations [50,51], as well as off-target bypass mechanisms [31]. In this setting, serial monitoring by circulating tumor DNA or repeat biopsy at progression may help define the next therapeutic strategy or support biomarker-directed trial enrollment. When selective RET inhibitors are unavailable, contraindicated, or no longer effective, multikinase inhibitors such as vandetanib and cabozantinib remain clinically important options, although they are not selected by a single predictive biomarker in routine practice [52,53]. Overall, genomic information is clinically meaningful in thyroid cancer only when interpreted in relation to tumor lineage, differentiation state, disease tempo, and the specific decision under consideration. In some settings, genomic testing changes treatment directly; in others, it refines risk, explains resistance, or identifies candidates for clinical trials. The next challenge is to determine whether functional molecular layers beyond driver genotype add reproducible and decision-relevant information to this framework. Figure 1 summarizes the major driver events, progression-associated alterations, resistance mechanisms, and matched targeted therapies across the principal thyroid cancer subtypes. Table 1 summarizes clinically established therapies in thyroid cancer and indicates which are biomarker-selected and which are selected primarily by histology, disease state, and progression pattern.

## 3. Functional Molecular Layers Beyond Driver Genotype

Driver genotype identifies important therapeutic opportunities in thyroid cancer, but it does not fully determine treatment sensitivity, resistance, or clinical behavior. Tumors with similar initiating alterations may differ substantially in differentiation state, pathway output, immune context, and capacity to respond to therapy. The clinical relevance of functional molecular layers therefore lies not in molecular complexity alone, but in whether they improve a defined decision beyond standard clinical, pathological, and genomic information. This section focuses only on non-genomic or higher-order molecular features that either inform a clinically meaningful decision or provide a well-supported mechanistic explanation for a phenotype already relevant to management. Exploratory associations without clear decision relevance are not catalogued in detail.

### 3.1. Chromatin Remodeling and Epigenetic Constraint on Differentiation

A major limitation of genotype-based prediction in thyroid cancer is that driver mutations do not fully explain whether a tumor retains a therapeutically usable thyroid-lineage program. In radioiodine-refractory disease, loss of differentiation may arise not only from signaling-mediated suppression of thyroid-specific genes, but also from more stable epigenetic and chromatin-level constraints that limit re-expression of iodine-handling machinery even when upstream oncogenic pathways are inhibited [21,58,59].

Among the most clinically relevant findings in this area is disruption of the SWI/SNF chromatin-remodeling complex. Loss-of-function alterations affecting subunits such as *ARID1A*, *SMARCA4,* and *PBRM1* are enriched in advanced and anaplastic thyroid cancers and impair chromatin accessibility at thyroid-lineage loci, including genes required for iodine uptake and organification [21]. At the same time, these changes favor a more mesenchymal, proliferative, and treatment-resistant tumor state. This has a direct practical implication: SWI/SNF disruption provides a mechanistic explanation for why some MAPK-driven tumors fail to recover radioiodine avidity despite pathway inhibition. Its clinical value currently lies in interpreting redifferentiation failure or reduced candidacy for redifferentiation strategies, rather than in serving as a stand-alone selection assay [21].

Other epigenetic mechanisms are biologically important but remain less mature clinically. Histone regulatory changes, including loss of *SETD2* and dysregulation of EZH2-mediated chromatin repression, have been associated with aggressive thyroid cancer biology and with impaired maintenance of differentiated cellular identity [9,19,60]. These observations support the concept that stable epigenetic fixation may limit reversibility of dedifferentiation and provide a rationale for combining epigenetic modulators with MAPK-pathway inhibition. However, such strategies remain preclinical or early translational, and no histone-based biomarker is currently validated to guide routine patient selection.

DNA methylation studies have likewise identified patterns associated with recurrence, metastasis, and dedifferentiation, but individual methylation markers have not reached sufficient reproducibility or clinical validation for stand-alone use [58,59]. At present, chromatin and epigenetic alterations are best interpreted as mechanistic modifiers of therapeutic vulnerability, particularly in relation to redifferentiation and resistance, rather than as routine decision tools.

### 3.2. Transcriptomic, Proteomic, and Metabolic Layers

Transcriptomic profiling is clinically relevant because it captures the biological output of genomic drivers and provides a functional readout of tumor state. In follicular-cell-derived thyroid cancers, one of the clearest examples is the distinction between BRAF-like and RAS-like transcriptional programs. These differ not only in MAPK output, but also in thyroid differentiation score, thereby linking driver genotype to the preservation or loss of thyroid-lineage function [16]. Expression of *SLC5A5*, *TG*, *TPO*, and *TSHR* is therefore informative not simply because these genes characterize differentiated thyroid cells, but because their coordinated expression reflects how intact the iodine-handling program remains and whether redifferentiation-based therapy is biologically plausible [9,16].

In PDTC and ATC, transcriptomic profiling further demonstrates that dedifferentiation is not binary but graded. More aggressive tumors show coordinated loss of thyroid-specific gene expression together with gain of proliferative, inflammatory, epithelial–mesenchymal, and cell-cycle-associated programs [9,19]. In this context, transcriptomic readouts may help distinguish tumors that remain lineage-constrained from those that have entered more plastic, inflammatory, or proliferative states that are less likely to respond to thyroid-specific therapeutic strategies.

Proteomic and phosphoproteomic profiling adds a complementary layer of information because therapeutic dependence is ultimately mediated by protein abundance, activation state, localization, and network activity rather than by DNA sequence alone. A kinase alteration may be present genomically but no longer represent the dominant functional dependency at the time of treatment selection. Proteogenomic studies in advanced differentiated, poorly differentiated, and anaplastic thyroid cancers have identified pathway-level vulnerabilities not fully resolved by genomic analysis alone, particularly in relation to MAPK, PI3K-AKT, stress-response, and cell-cycle programs [61,62]. Even so, these approaches remain investigational. Their future value will depend on whether they reproducibly improve prediction of redifferentiation potential, kinase-inhibitor sensitivity, rational combination therapy, or resistance mechanisms beyond established clinicopathological and genomic variables.

Metabolic profiling is most clearly informative in oncocytic carcinoma. In this subtype, recurrent mitochondrial DNA mutations involving complex I subunits provide a biological explanation for the oncocytic phenotype and support the concept of subtype-specific metabolic liability [44,45]. Integrative studies further suggest dependence on compensatory metabolic programs, including aerobic fermentation and redox adaptation, although these findings remain translational rather than actionable in routine care [44,45]. A further candidate found in oncocytic carcinoma is retention or duplication of chromosome 7 within an otherwise genome-wide loss-of-heterozygosity pattern, a feature proposed to mark more aggressive disease while preserving specific oncogenic loci [63]. This remains hypothesis-generating and not yet clinically deployable.

Outside oncocytic carcinoma, most metabolomic findings remain exploratory. Nevertheless, the clinically familiar “flip-flop” pattern, loss of radioiodine avidity with increased FDG uptake, illustrates that metabolic change can function as a practical surrogate of dedifferentiation and adverse biology. Although FDG-PET is not metabolomics in an analytical sense, it remains the most established example of clinically relevant metabolic state in thyroid cancer.

Longitudinal circulating tumor DNA analysis represents a special case. Although it samples genomic rather than transcriptomic or epigenetic information, it is functionally distinct because it enables serial, non-invasive assessment of evolving tumor biology. Its role in diagnosis, resistance monitoring, and progression assessment has already been discussed in Section 2, where it represents the most clinically mature dynamic molecular adjunct considered in this review.

By contrast, most non-coding RNA and extracellular-vesicle biomarkers remain exploratory. MicroRNAs, long non-coding RNAs, circular RNAs, and related signatures have been associated with recurrence, nodal spread, or aggressive behavior, especially in PTC, but they do not yet define a current clinical decision and therefore remain of mainly biological or discovery-stage interest [64,65,66,67,68,69,70,71,72,73].

### 3.3. Immune, Single-Cell, and Spatial Profiling

The tumor microenvironment becomes increasingly important as thyroid cancers progress toward dedifferentiated and treatment-resistant states. This is particularly relevant in ATC, where immune context may influence both disease behavior and response to systemic therapy. However, immune infiltration alone is not sufficient biomarkers. Tumors may appear inflamed yet remain therapeutically resistant because cytotoxic T cells are spatially excluded, functionally exhausted, or suppressed by macrophage-rich and stromal compartments [74,75,76].

ATC exhibits marked immune heterogeneity. A subset of tumors shows relatively “hot” immune phenotypes with increased expression of checkpoint-related molecules such as programmed death-ligand 1 (PD-L1), programmed death-ligand 2 (PD-L2), cytotoxic T-lymphocyte-associated protein 4 (CTLA-4), and T-cell immunoreceptor with Ig and ITIM domains (TIGIT), whereas others are profoundly immune-depleted [74]. These patterns are potentially trial-enabling for immunotherapy stratification, but they are not yet routine decision tools in thyroid-specific practice. A more specific mechanistic finding is the presence of macrophage-dominant immune states, including M2-polarized signatures and upregulation of the anti-phagocytic signal CD47 [75,76]. This identifies a biologically coherent immunosuppressive niche and suggests a druggable node, but the current relevance remains preclinical or early translational rather than standard-of-care [76].

A related but distinct immune-genomic relationship has been described in oncocytic carcinoma, where extensive loss of heterozygosity correlates inversely with immune infiltration [77]. The most genomically unstable tumors may therefore be the most immune-depleted, possibly because of reduced antigen-presentation capacity and lower effective immunogenicity. At the same time, PD-L1 expression may still be upregulated in selected cases and correlate with macrophage abundance, suggesting that global immune depletion does not necessarily exclude selective relevance of immune-checkpoint approaches [78].

Single-cell and spatial profiling technologies add clinically meaningful depth by resolving how malignant, immune, and stromal cell populations are organized within the tumor. These methods can identify lineage transitions, immune exclusion, stromal barriers, and heterogeneous malignant-cell states that bulk profiling may miss [79,80]. They are especially informative in anaplastic transformation, where small biopsies may under-sample biologically distinct tumor compartments. At present, however, their main value lies in mechanism discovery, resistance mapping, and biomarker development rather than in routine clinical decision-making.

The same caution applies to immune biomarkers such as PD-L1 expression, mismatch-repair deficiency, microsatellite instability, and tumor mutational burden. These may carry regulatory or tumor-agnostic therapeutic implications, but they are not thyroid-genotype-specific biomarkers and should not be assigned the same evidentiary meaning as lineage- or subtype-specific molecular determinants.

### 3.4. Host Factors

Among host-associated molecular layers, the microbiome is currently the least mature in relation to thyroid cancer management. Differences in gut and oral microbiota composition have been described in patients with thyroid cancer or thyroid nodules, and several mechanisms have been proposed, including effects on iodine availability, bile-acid metabolism, thyroid hormone handling, systemic inflammation, and immune tone [81,82,83,84]. These mechanisms are biologically plausible and may eventually prove relevant to treatment tolerance or host-tumor interaction.

However, the available evidence remains heavily confounded. Diet, iodine intake, antibiotic exposure, thyroid hormone replacement, proton-pump inhibitor use, gastrointestinal disease, surgery, and radioiodine therapy can all influence microbial composition independently of tumor biology [81,82,83]. Radioiodine treatment itself may alter oral microbiota, particularly in patients with xerostomia or salivary dysfunction [84]. Because current studies do not adequately control for this full set of confounders, the microbiome should not presently be regarded as a validated diagnostic, prognostic, or therapeutic biomarker in thyroid cancer.

Its most plausible future role may be as a modifier of treatment tolerance, levothyroxine absorption, mucosal toxicity, or immunotherapy responsiveness rather than as a primary tumor biomarker. For now, however, it remains exploratory and should be interpreted cautiously.

### 3.5. Clinical Integration of Functional Molecular Layers

Taken together, these functional molecular layers refined the concept of therapeutic vulnerability in thyroid cancer. Chromatin and epigenetic states help explain why some tumors remain stably dedifferentiated despite pathway inhibition. Transcriptomic and proteomic profiling define the biological output of drivers and the degree to which thyroid-lineage function, kinase dependency, or proliferative escape remains active. Metabolic features, particularly in oncocytic carcinoma, reveal subtype-specific dependencies that are not apparent from canonical driver analysis alone. Immune, single-cell, and spatial approaches clarify the architecture of resistance and may identify biologically meaningful subgroups for future trial design. Host-associated factors such as the microbiome remain preliminary and have no current decision-making role.

The clinical value of these layers will depend less on their novelty than on their ability to improve a defined decision. Prediction of redifferentiation benefit, for example, may require integration of driver genotype, thyroid differentiation score, NIS expression or localization, prior radioiodine imaging, and treatment history. Prediction of immunotherapy benefit may require joint assessment of tumor genotype, checkpoint expression, myeloid abundance, immune spatial organization, and prior systemic therapy. Similarly, prediction of targeted-therapy resistance may require baseline genotype together with dynamic reassessment at progression.

Selected functional molecular layers and their current evidence maturity are summarized in Table 2. Because most remain translational, trial-enabling, or exploratory rather than routine clinical tools, the table should be interpreted as an evidence map rather than as a list of biomarkers ready for universal implementation.

## 4. Computational Integration and Artificial Intelligence in Precision Thyroid Oncology

**Figure 2:**
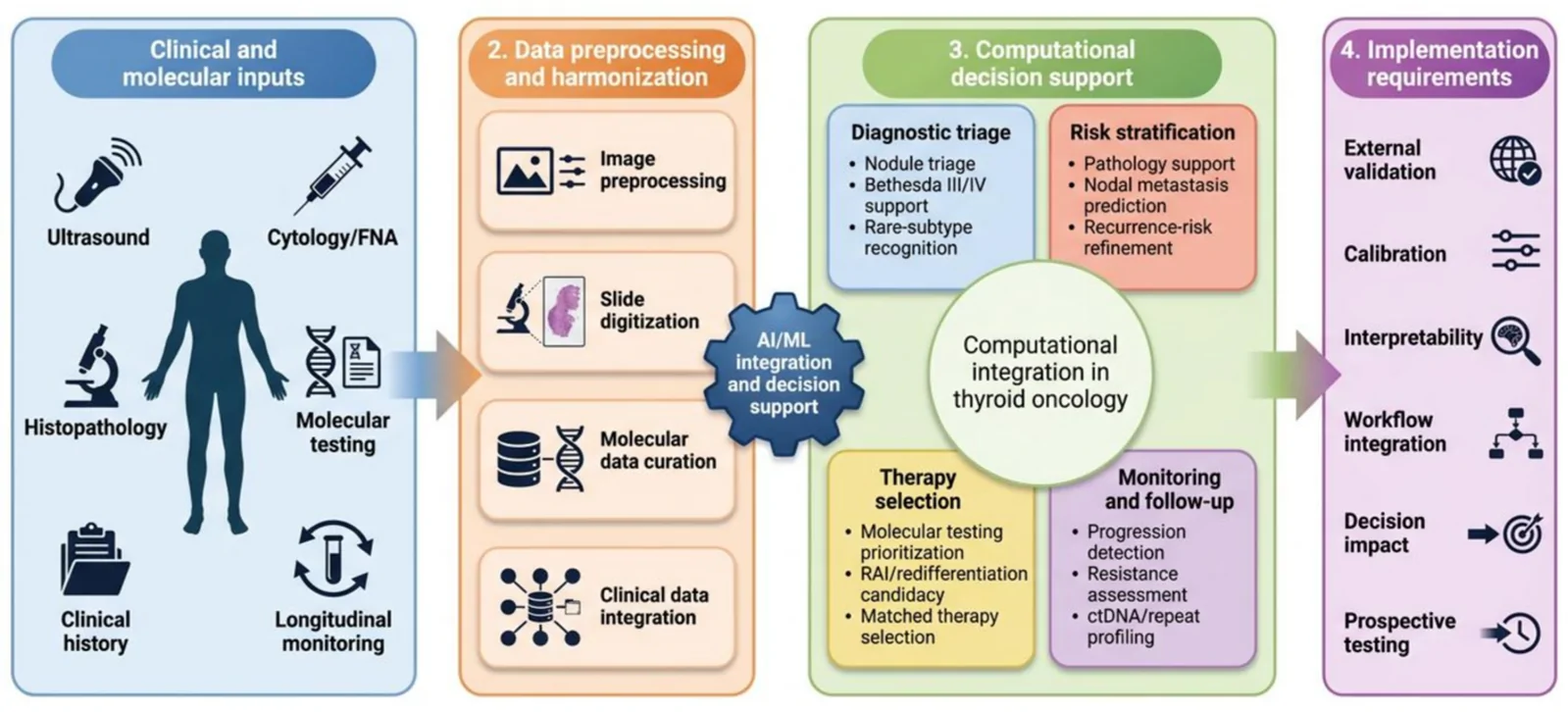
Computational integration across key decision points in precision thyroid oncology. Clinical, cytological, imaging, histological, treatment-history, and molecular data can be integrated to support thyroid cancer management across diagnosis, risk stratification, therapy selection, progression monitoring, and follow-up. The workflow shown includes multidisciplinary evaluation, tissue or liquid sampling, multimodal data generation, computational integration, and decision support. Clinical implementation requires calibration, external validation, interpretability, workflow integration, and demonstration of decision impact.

Clinically useful information in thyroid cancer is distributed across ultrasound images, cytology, histopathology, molecular classifiers, genomic reports, treatment history, and longitudinal follow-up data. Computational methods are increasingly proposed to integrate these layers into clinically actionable risk estimates. Their relevance to precision thyroid oncology, however, should not be assumed on the basis of algorithmic sophistication or retrospective discrimination alone. A useful model is one that improves a clearly defined clinical decision, such as whether to perform fine-needle aspiration (FNA), proceed to diagnostic surgery, intensify surveillance, prioritize molecular testing, or select matched systemic therapy. This standard is particularly important in thyroid cancer, where both overtreatment of indolent nodules and delayed recognition of aggressive disease remain major clinical risks. Most published thyroid artificial intelligence (AI) studies remain focused on ultrasound-based benign–malignant nodule classification. In a systematic review and meta-analysis restricted to externally validated datasets, Gatta et al. [85] analyzed 27 studies including 146,332 patients and more than 600,000 ultrasound images. The pooled diagnostic performance was strong, with sensitivity of 87%, specificity of 83%, and a summary area under the curve (AUC) of 91.9%. These findings support ultrasound AI as a potentially useful adjunct for nodule triage and reduction in operator dependence, but not as an autonomous diagnostic tool. Importantly, many included cohorts were surgically, or cytologically enriched, low-risk nodules managed conservatively were underrepresented, and geographic representation was limited, with a predominance of Asian cohorts. The clinically relevant question is therefore not whether ultrasound AI performs well retrospectively, but whether it can safely reduce unnecessary FNA or diagnostic surgery without increasing missed clinically meaningful cancers. Rare-subtype recognition represents a distinct computational challenge because most imaging models are trained predominantly on benign nodules and papillary thyroid carcinoma. Dai et al. [86] addressed this problem using Tiger, a text-guided diffusion model trained on 186,812 ultrasound images from 40,845 patients. In this study, feature-guided synthetic augmentation improved the AUC for benign-versus-follicular thyroid carcinoma classification from 0.736 to 0.844 and for benign-versus-medullary thyroid carcinoma from 0.752 to 0.823. In an external test set of anaplastic thyroid carcinoma, the AUC improved from 0.769 to 0.866. In addition, physicians judged Tiger-generated images to be realistic in 92.2% of cases. These results suggest that clinically informed synthetic augmentation may help train models for rare subtypes, but the generated images themselves do not constitute clinical validation. Their current value lies in improving training data diversity rather than in direct decision support. AI applications in pathology may prove especially relevant because diagnostic uncertainty often persists after imaging. Almagor et al. [87] combined spectral imaging of hematoxylin-and-eosin-stained thyroid sections with nuclear segmentation and a random forest classifier in a study of 41 thyroid resection specimens comprising 208,704 nuclei. Using spectral, morphologic, and spatial nuclear features, the model achieved F1/AUC values of 0.891/0.931 for papillary thyroid carcinoma, 0.821/0.801 for follicular variant papillary thyroid carcinoma, and 0.825/0.858 for non-invasive follicular thyroid neoplasm with papillary-like nuclear features (NIFTP). This study is notable because it provides a relatively interpretable nucleus-level approach to pathology support, but it remains limited by small sample size, the need for dedicated imaging hardware, and the lack of large multicenter prospective validation. Similar limitations apply to many cytology-based models, which often show promising retrospective performance but remain constrained by slide digitization requirements, annotation burden, preparation variability, and limited external testing. Multimodal and vision-language systems may have greater near-term value as supervised decision-support tools rather than independent diagnostic models. Yao et al. [88] developed ThyGPT using 511,620 ultrasound images, 49,733 reports, and guideline-based diagnostic knowledge from 59,406 patients. In the study setting, use of the system improved radiologist AUC for thyroid nodule risk assessment from 0.805 to 0.908. The ThyGPT-assisted strategy was also estimated to reduce the FNA rate from 64.2% to 23.3% while lowering missed malignancies from 11.6% to 5.3%. In addition, report-error detection accuracy reached 0.905, and radiologist error detection improved from 0.764 to 0.962 with model assistance. These findings support the potential role of multimodal AI in triage and quality control, but also highlight the need for continued human supervision, because generative systems may produce biased, uncertain, or clinically inappropriate outputs if applied beyond their training context. Computational prediction may also assist surgical planning. Shen et al. [89] developed LLNM-Net, a multimodal transformer trained on 39,451 patients across seven centers, integrating ultrasound images, radiology reports, demographic variables, and pathology data to predict lateral lymph node metastasis. In the external test set, the model achieved an AUC of 0.944 and an accuracy of 0.847 for lateral lymph node metastasis prediction, while high-risk nodal disease was identified with an AUC of 0.971. Tumor location was an especially influential feature, with nodules located within 0.25 cm of the thyroid capsule associated with a greater than 72% probability of lateral lymph node metastasis. Such models could support decisions regarding lateral-neck ultrasound, targeted nodal FNA, and surgical planning, but prospective testing is still needed to determine whether their use improves outcomes without increasing unnecessary intervention. The most relevant studies in a multi-omics context are those that genuinely integrate molecular with clinical data. Zhou et al. [90] evaluated this approach in medullary thyroid carcinoma using 482 samples from 452 patients collected across 10 centers. Their study integrated clinical, genomic, proteomic, and ubiquitinomic information using multi-omics random forest modeling and non-negative matrix factorization. An integrated model based on 18 proteins and 2 clinical variables predicted structural recurrence with an AUC of 0.87 in an independent test cohort and 0.77 in an external validation dataset. Proteomic subtyping also separated patients into poor- and favorable-prognosis groups, supporting postoperative recurrence-risk refinement in medullary thyroid carcinoma. This study is particularly relevant to the theme of this review because it moves beyond imaging alone and attempts true molecular-clinical integration; however, it remains retrospective and tissue-based, and its incremental value over established clinical predictors must still be confirmed prospectively. Taken together, these studies show that the field is evolving from retrospective image classification toward decision-linked systems for nodule triage, rare-subtype recognition, pathology support, nodal risk prediction, report quality control, and recurrence modeling. They also illustrate an important imbalance: most current AI models in thyroid cancer still operate on imaging or pathology data alone, whereas relatively few integrate molecular information in a way that is directly relevant to multi-omics-guided management. Figure 2 summarizes how imaging, pathology, molecular, and clinical layers may converge across key management decisions, and Table 3 provides a structured overview of representative computational studies, including their data sources, tasks, performance metrics, and clinical implications.

Implementation standards for these tools should remain strict. High AUC is insufficient if a model is poorly calibrated, population-specific, opaque, difficult to integrate into workflow, or unable to improve clinical decision-making. External validation is essential because ultrasound devices, image acquisition protocols, cytology thresholds, pathology workflows, molecular-test availability, and population structure vary across institutions and regions. Interpretability must also be clinically meaningful: clinicians need explanations linked to recognizable features such as nodule margins, echogenicity, calcification, cytological class, molecular result, genotype, disease tempo, or toxicity risk. Future computational studies should therefore be judged by decision-relevant endpoints rather than by retrospective accuracy alone. The most meaningful benchmarks include reduction in unnecessary FNA or surgery, safe expansion of surveillance, earlier recognition of rare aggressive subtypes, faster access to matched therapy in advanced disease, improved prediction of radioiodine refractoriness or redifferentiation benefit, earlier identification of resistance, and reduced severe toxicity. In thyroid cancer, computational integration is clinically valuable only when it links multimodal data to decisions that measurably improve patient management.

## 5. Implementation Barriers and Future Directions

Despite major advances in molecular characterization, the clinical translation of genomic, multi-omics, and computational tools in thyroid cancer remains uneven. The strongest evidence supports selective use in settings where the result can change management: molecular testing of Bethesda III/IV nodules [10,91,92], rapid genomic profiling in anaplastic thyroid carcinoma [8,33], and tumor testing in advanced radioiodine-refractory, metastatic, or medullary thyroid cancer when results can identify an approved targeted therapy or a relevant clinical trial [33,93]. In contrast, broad multi-omics profiling has limited immediate value in many low-risk differentiated thyroid cancers, where outcomes are already excellent and management is largely guided by established clinicopathological risk assessment [10,92]. This distinction is central to implementation: molecular testing is justified when it changes a diagnostic, surgical, or therapeutic decision, and remains investigational when it adds biological detail without a management consequence [33,92].

A first major barrier is evidentiary maturity. Many proposed biomarkers derived from methylation, transcriptomic, proteomic, metabolomic, immune, single-cell, spatial, and microbiome studies are biologically compelling but have not been validated prospectively in treatment-linked cohorts. Most studies remain retrospective, single-center, surgically enriched, technically heterogeneous, and underpowered for clinically meaningful endpoints. This is particularly problematic in thyroid cancer because many molecularly interesting phenotypes occur in uncommon subtypes, rare progression states, or highly selected advanced-disease cohorts. Future studies must therefore move beyond descriptive associations and test whether proposed biomarkers improve diagnosis, recurrence-risk prediction, therapy selection, resistance interpretation, or toxicity management beyond established clinical, pathological, and genomic variables [33,92]. A second barrier concerns sampling, spatial heterogeneity, and temporal evolution. Molecular interpretation may differ substantially depending on whether testing is performed on primary tumor tissue, metastatic lesions, archival material, repeat biopsy samples, or circulating tumor DNA [29,30,31,33]. Archival thyroidectomy tissue may fail to capture later progression-associated events in advanced disease. Small biopsies, particularly in ATC, may under-sample spatially heterogeneous malignant and immune compartments [8,29,33]. Conversely, repeat tissue sampling may be impractical because of rapid clinical deterioration, inaccessible lesions, or procedural risk. Circulating tumor DNA offers an attractive complement because it allows non-invasive longitudinal monitoring, but it also has limitations, including reduced sensitivity in low-volume disease, dependence on assay design, and incomplete resolution of spatially distinct tumor clones [30,31]. In practice, tissue, repeat biopsy, and liquid biopsy should be viewed as complementary rather than interchangeable sources of molecular information. A third barrier is assay standardization and interpretive consistency. Even when a biomarker is biologically plausible, clinical adoption requires harmonized pre-analytical handling, reproducible analytical performance, validated reporting thresholds, and clear interpretation rules [33,93,94]. This challenge applies across modalities, from gene panels and molecular classifiers to phosphoproteomic, spatial, and computational assays. For multi-omics to become clinically credible, laboratories must demonstrate not only technical validity but also reproducibility across platforms and institutions [33,93]. Molecular tumor boards may facilitate interpretation of complex results, but recommendations should remain evidence-ranked, auditable, and explicitly linked to a management question rather than driven by biological plausibility alone [33]. Cost, reimbursement, and health-system feasibility represent a fourth barrier. In indeterminate thyroid nodules, the economic value of molecular testing depends heavily on local malignancy prevalence, assay price, surgical cost, complication risk, expert cytopathology availability, and whether testing actually changes the decision to operate or only the extent of surgery [91,92]. A highly accurate assay is not automatically cost-effective if surgery would have been performed regardless of the result. In advanced disease, the calculation is different: the value of genomic profiling depends on whether it enables access to effective matched therapy, avoids ineffective treatment, or guides trial enrollment [93,95]. The high cost of targeted therapies, regional differences in reimbursement, and the need for sustained toxicity management all influence real-world utility [93,95]. Broad profiling is therefore most defensible when it identifies a treatment option that is both clinically appropriate and realistically accessible to the patient. Turnaround time is a fifth practical limitation. In anaplastic thyroid carcinoma, clinically meaningful molecular results must be available rapidly because actionable alterations may affect urgent first-line management [8,33]. Delayed profiling may miss a narrow therapeutic window. In more slowly progressive radioiodine-refractory differentiated thyroid cancer, broader profiling may be more feasible and can support sequencing decisions, redifferentiation strategies, or trial referral [33,93]. The required speed, depth, and breadth of testing should therefore be adapted to disease tempo and decision urgency rather than applied uniformly across all thyroid cancers. Implementation challenges also extend to computational models. Many AI studies report strong retrospective performance, especially in ultrasound classification, but adoption requires more than high area under the curve [85,96]. Models must be calibrated to local disease prevalence, externally validated across institutions and devices, interpretable to clinicians, robust to workflow variation, and tested prospectively against decision-impact endpoints [96]. A model trained on surgically enriched cohorts may overestimate malignancy risk in surveillance populations, and a model trained mainly on papillary thyroid carcinoma may underperform in medullary, follicular, or anaplastic disease [85,88,89]. Computational tools should therefore remain decision-support systems unless they have demonstrated real-world clinical benefit under prospective conditions [96]. Equity and generalizability remain unresolved and deserve explicit attention. Many genomic, multi-omics, and AI datasets are derived from limited populations and health-care systems, with underrepresentation of geographic, ethnic, and resource-diverse settings [93,95]. Yet thyroid cancer incidence, iodine exposure, imaging practice, cytology thresholds, surgical preferences, access to testing, and availability of targeted therapies all vary internationally. A biomarker or model validated in one context may not retain the same predictive value in another [93,95]. Responsible implementation therefore requires multicenter and multiethnic validation, transparent reporting of ancestry and geography, and attention to affordability, laboratory capacity, data governance, and post-test access to matched therapies.

Future progress in precision thyroid oncology should be measured by decision impact rather than by molecular breadth alone. A new assay, integrated signature, or computational model should be judged by whether it reduces unnecessary surgery, improves prevalence-aware triage of indeterminate nodules, strengthens postoperative risk stratification, identifies patients likely to benefit from radioiodine or redifferentiation therapy, accelerates access to effective matched systemic treatment, detects resistance earlier, reduces toxicity, or improves survival and quality of life [33,92,96]. Without such evidence, even technically sophisticated biomarkers should remain investigational. The priority is therefore not simply broader testing, but disciplined implementation of molecular and computational tools that are validated, feasible, equitable, and genuinely management changing.

## 6. Conclusions

Thyroid cancer now has a detailed molecular map, but the central challenge is no longer identifying additional alterations for their own sake. It is determining which molecular findings define a clinically meaningful vulnerability and alter management across diagnosis, treatment, resistance monitoring, and follow-up. The clearest examples are already embedded in practice: molecular classifiers can refine management of cytologically indeterminate nodules, *BRAF* V600E identifies a targetable subset of anaplastic thyroid carcinoma, *RET* alterations guide selective RET inhibition in medullary and fusion-positive disease, and *NTRK* fusions identify candidates for TRK inhibitors. Beyond these established applications, the field is moving toward a broader but more demanding conception of precision thyroid oncology. In differentiated thyroid cancer, clinically relevant vulnerability depends not only on driver genotype but also on preservation of thyroid-lineage function and the possibility of restoring therapy sensitivity in selected tumors. In advanced disease, response and resistance are shaped by cooperating genomic events, lineage plasticity, immune context, and metabolic adaptation. Multi-omics approaches have expanded understanding of these states, but most proposed biomarkers remain translational, trial-enabling, or exploratory rather than ready for routine care.

Three priorities should now guide the field. First, prospective studies are needed to determine where molecular testing changes management in a measurable way, particularly in indeterminate nodules, redifferentiation strategies, and advanced disease. Second, longitudinal precision oncology must be strengthened through studies that define when repeat biopsy or circulating tumor DNA monitoring improves resistance interpretation and therapeutic adaptation. Third, integrated multi-omics and computational models must be evaluated not only for discrimination, but for calibration, external validity, implementation feasibility, cost-effectiveness, and equity across diverse health-care settings. Progress in thyroid cancer precision medicine will therefore depend less on the number of available biomarkers than on the discipline with which they are validated and applied. Molecular information should be considered clinically meaningful only when it improves a defined decision, adds value beyond existing variables, and can be implemented responsibly in routine care. That standard is demanding, but it is also what will determine whether genomics and multi-omics genuinely improve outcomes for patients with thyroid cancer.

## Figures and Tables

**Table 1:** Clinically established therapies in thyroid cancer and their molecular context.

Therapy	Principal Clinical Setting	Molecular Requirement	Evidence Tier	Clinical Decision Informed	Regulatory Status	References
Levothyroxine (TSH suppression)	Resected DTC	None (endocrine suppression)	Standard of care	Adjuvant management; no genomic selection	Guideline standard	[7,10]
Radioactive iodine (^131^I)	Intermediate–high-risk DTC; RAI-avid metastatic disease	Preserved thyroid differentiation/NIS function	Standard of care	Adjuvant/therapeutic use, RAI-avid disease only	Guideline standard	[6,7,10]
Sorafenib	Progressive RAIR-DTC	None validated for selection	Standard of care	First-line systemic option, RAIR-DTC	FDA approved	[54]
Lenvatinib	Progressive RAIR-DTC	None validated for selection	Standard of care	Preferred first-line systemic option	FDA approved	[55]
Cabozantinib	RAIR-DTC, post-VEGFR therapy	None validated for selection	Standard of care	Second-line or later	FDA approved	[56]
Vandetanib	Progressive/symptomatic MTC	None mandatory	Standard of care	Used when RET-selective agents unsuitable	FDA approved	[52]
Cabozantinib	Progressive MTC	None mandatory	Standard of care	Used when RET-selective agents unsuitable	FDA approved	[53]
Selpercatinib	Advanced/metastatic MTC; RET fusion+ DTC	RET mutation or fusion	Standard of care	Biomarker-selected, first-line or later	FDA approved	[25,48]
Pralsetinib	Advanced/metastatic MTC; RET fusion+ DTC	RET mutation or fusion	Standard of care	Biomarker-selected systemic therapy	FDA approved	[49]
Dabrafenib + trametinib	Unresectable/metastatic ATC	BRAF p.V600E	Standard of care	Biomarker-selected first-line, when feasible	FDA approved	[22]
Larotrectinib	Advanced thyroid cancer	NTRK fusion	Standard of care	Tumor-agnostic biomarker-selected therapy	FDA approved	[23]
Entrectinib	Advanced thyroid cancer	NTRK fusion	Standard of care	Tumor-agnostic biomarker-selected therapy	FDA approved	[57]

Abbreviations: ATC, anaplastic thyroid carcinoma; DTC, differentiated thyroid cancer; MTC, medullary thyroid carcinoma; NIS, sodium/iodide symporter; RAI, radioactive iodine; RAIR-DTC, radioiodine-refractory differentiated thyroid cancer; RET, RET proto-oncogene; TSH, thyroid-stimulating hormone; VEGFR, vascular endothelial growth factor receptor. Regulatory status may vary across jurisdictions.

**Table 2:** Functional molecular layers beyond driver genotype in thyroid cancer and their current evidence maturity.

Molecular Layer	Marker or Readout	Thyroid Cancer Context	Biological Relevance	Potential Clinical Relevance	Evidence Maturity	Main Limitation	References
DNA methylation	Global and recurrence-associated methylation signatures	DTC; advanced/dedifferentiated thyroid cancer	Epigenetic loss of lineage identity; metastasis/dedifferentiation	Mechanistic modifier of redifferentiation resistance and tumor progression	Translational	No validated standalone selection assay	[58,59]
Chromatin remodeling	SWI/SNF disruption	Advanced follicular-cell-derived thyroid cancer	Loss of thyroid differentiation program	Mechanistic rationale for redifferentiation resistance	Preclinical/translational	Requires clinical treatment-linked validation	[21]
Histone regulation	EZH2 overexpression/altered H3K27me3 regulation	ATC and aggressive thyroid cancer	Repressive chromatin state; aggressive phenotype	Rationale for epigenetic therapy and redifferentiation research	Preclinical/translational	No established thyroid cancer indication	[60]
Thyroid-lineage transcriptome	Thyroid differentiation score (*SLC5A5*, *TG*, *TPO*, *TSHR*)	PTC, PDTC, ATC	Composite readout of thyroid-lineage integrity and iodine-handling capacity	Candidate selection for redifferentiation strategies	Translational	Not standardized for treatment selection	[16,17]
Thyroid-lineage protein localization	NIS membrane localization	DTC, RAIR-DTC	Functional iodine uptake	Candidate predictor of radioiodine responsiveness	Translational	Requires validated assay and tissue availability	[17]
MAPK transcriptional output	BRAF-like expression state	PTC	High MAPK output; lower thyroid differentiation	Risk and radioiodine biology refinement	Translational	Requires assay harmonization	[16]
MAPK transcriptional output	RAS-like expression state	PTC, follicular-patterned tumors	Lower MAPK output; more differentiated phenotype	Molecular classification and biologic stratification	Translational	Not directly treatment-selective	[16]
Proteome/phosphoproteome	Pathway-activity signatures	Advanced DTC, PDTC, ATC	Functional pathway dependence beyond DNA sequence	Candidate refinement of kinase-inhibitor sensitivity and resistance mapping	Translational	Incremental clinical value not yet prospectively validated	[61,62]
Metabolome/genomic instability	Complex I mtDNA mutations; chromosome 7 duplication	Oncocytic carcinoma	Explains oncocytic phenotype; metabolic adaptation; genomic instability	Candidate prognostic marker and metabolic vulnerability	Translational	No validated therapeutic selection role	[44,45,63]
Genomic (liquid biopsy)	Circulating tumor DNA	Advanced PTC, PDTC, ATC, MTC	Serial assessment of evolving tumor genotype	Resistance/progression monitoring and trial selection	Trial-enabling	Limited sensitivity in low-volume disease; assay standardization needed	[29,30,31]
Immune/spatial	ATC immune landscape; 78-gene M2-macrophage signature; CD47	ATC	Immune heterogeneity; macrophage-rich immunosuppressive niche	Candidate immunotherapy stratification; CD47 as druggable target	Trial-enabling/preclinical	Not validated as routine thyroid-specific biomarkers	[74,75,76]
Immune-genomic instability	Loss of heterozygosity vs. immune infiltration	Oncocytic carcinoma	Links genomic instability to immune depletion	Candidate immune-stratification marker	Translational	Requires independent validation and treatment-linked correlation	[77,78]
Host factor	Gut/oral microbiome composition	Thyroid nodules/cancer	Potential modifier of inflammation, hormone handling, and treatment tolerance	No current decision-making role	Exploratory	Strong confounding and lack of causal evidence	[81,82,83,84]

Abbreviations: ATC, anaplastic thyroid carcinoma; DTC, differentiated thyroid cancer; MTC, medullary thyroid carcinoma; PDTC, poorly differentiated thyroid carcinoma; PTC, papillary thyroid carcinoma; RAIR-DTC, radioiodine-refractory differentiated thyroid cancer; NIS, sodium/iodide symporter; mtDNA, mitochondrial DNA; H3K27me3, trimethylation of histone H3 lysine 27.

**Table 3:** Representative computational studies relevant to precision thyroid oncology.

Study	Data	Clinical Task	Model Type	Main Result	Clinical Implication
[85]	27 studies; 146,332 patients; >600,000 ultrasound images	Benign–malignant nodule classification	Ultrasound AI	Sensitivity 87%; specificity 83%; summary AUC 91.9%	Supports AI as an adjunct for nodule triage, but not autonomous diagnosis
[86]	40,845 patients; 186,812 ultrasound images	Rare thyroid cancer subtype augmentation	Text-guided diffusion model	AUC improved for FTC 0.736→0.844, MTC 0.752→0.823, external ATC 0.769→0.866	Synthetic augmentation can improve rare-subtype learning; clinical validation remains required
[88]	59,406 patients; 511,620 ultrasound images; 49,733 reports	Nodule risk assessment, FNA triage, report-error detection	Multimodal GPT/AIGC-CAD	Radiologist AUC 0.805→0.908; FNA rate 64.2%→23.3%; missed malignancy 11.6%→5.3%; report-error detection 0.905	Vision-language AI may support triage and report quality control under human supervision
[89]	39,451 patients from 7 centers	Lateral lymph node metastasis prediction	Multimodal transformer	External AUC 0.944; high-risk nodal disease AUC 0.971	May guide lateral-neck ultrasound, FNA and surgical planning
[87]	41 thyroid resection specimens; 208,704 nuclei	Pathology support for PTC, FVPTC and NIFTP	Spectral imaging, nuclear segmentation, random forest	F1/AUC: PTC 0.891/0.931; FVPTC 0.821/0.801; NIFTP 0.825/0.858	Interpretable nuclear-level pathology support; requires larger validation and dedicated hardware
[90]	482 MTC samples from 452 patients across 10 centers	MTC recurrence prediction and proteomic subtyping	Multi-omics random forest; NMF	Integrated model AUC 0.87 (independent test), 0.77 (external dataset)	Supports recurrence-risk refinement in MTC, but remains retrospective and tissue based

Abbreviations: AI, artificial intelligence; AUC, area under the curve; ATC, anaplastic thyroid carcinoma; FNA, fine-needle aspiration; FTC, follicular thyroid carcinoma; FVPTC, follicular variant papillary thyroid carcinoma; GPT, generative pre-trained transformer; AIGC-CAD, AI-generated content/computer-aided diagnosis; MTC, medullary thyroid carcinoma; NIFTP, non-invasive follicular thyroid neoplasm with papillary-like nuclear features; NMF, non-negative matrix factorization; PTC, papillary thyroid carcinoma. The arrow (→) indicates a change in the reported metric from baseline (before) to post-intervention or post-optimization (after) values, as specified in each corresponding study.

## Data Availability

No new data were created or analyzed in this study. Data sharing is not applicable to this article.

## Abbreviations

AI	artificial intelligence
ALK	anaplastic lymphoma kinase
ATC	anaplastic thyroid carcinoma
AUC	area under the curve
BRAF	B-Raf proto-oncogene, serine/threonine kinase
CTLA-4	cytotoxic T-lymphocyte-associated protein 4
ctDNA	circulating tumor DNA
DHGTC	differentiated high-grade thyroid carcinoma
DTC	differentiated thyroid cancer
EGFR	epidermal growth factor receptor
FDA	Food and Drug Administration
FNA	fine-needle aspiration
FTC	follicular thyroid carcinoma
FVPTC	follicular variant papillary thyroid carcinoma
H&E	hematoxylin and eosin
ICI	immune checkpoint inhibitor
MAPK	mitogen-activated protein kinase
MEK	mitogen-activated protein kinase kinase
MTC	medullary thyroid carcinoma
NIFTP	non-invasive follicular thyroid neoplasm with papillary-like nuclear features
NIS	sodium/iodide symporter
NMF	non-negative matrix factorization
NTRK	neurotrophic tyrosine receptor kinase
PD-L1	programmed death-ligand 1
PD-L2	programmed death-ligand 2
PDTC	poorly differentiated thyroid carcinoma
PI3K–AKT	phosphatidylinositol 3-kinase–AKT pathway
PTC	papillary thyroid carcinoma
RAI	radioactive iodine
RAIR	radioiodine-refractory
RAIR-DTC	radioiodine-refractory differentiated thyroid cancer
RET	RET proto-oncogene
SLC5A5	solute carrier family 5 member 5
SWI/SNF	switch/sucrose non-fermentable chromatin-remodeling complex
TCGA	The Cancer Genome Atlas
TERT	telomerase reverse transcriptase
TG	thyroglobulin
TIGIT	T-cell immunoreceptor with Ig and ITIM domains
TPO	thyroid peroxidase
TP53	tumor protein p53
TRK	tropomyosin receptor kinase
TSH	thyroid-stimulating hormone
TSHR	thyroid-stimulating hormone receptor
VEGFR	vascular endothelial growth factor receptor
WHO	World Health Organization
